# Could fluvoxamine keep COVID-19 patients out of hospitals and intensive care units?

**DOI:** 10.3325/cmj.2021.62.95

**Published:** 2021-02

**Authors:** Robert Marčec, Robert Likić

**Affiliations:** 1 University of Zagreb School of Medicine, Zagreb, Croatia; 2 Unit for Clinical Pharmacology, Department of Internal Medicine, Clinical Hospital Center Zagreb, Zagreb, Croatia *robert.likic@mef.hr*

Since December 2019, when the first cases of coronavirus disease 2019 (COVID-19) were reported in Wuhan, China, SARS-CoV-2 has caused a global pandemic, with more than 75 million cases and over 1.6 million deaths worldwide (1). The international community has invested enormous resources in the development of safe and effective vaccines, which were registered and approved for widespread use in record time. Nevertheless, limited production capacities and vaccine hesitancy could impede a global post-COVID-19 recovery, particularly in developing countries.

Until the end of 2021, two to four billion vaccine doses may be produced globally, if most vaccine candidates become licensed for human use. Almost 50% of the projected worldwide production has already been pre-ordered by wealthy countries – the UK pre-ordered staggering five doses per capita (2). Although rich countries may have more than enough vaccine doses available to reach herd immunity levels in their populations and initiate the process of post-pandemic recovery, vaccine hesitancy could prolong the time needed to reach sufficient immunity levels, or even prevent it altogether, particularly in parts of the USA (3) and Europe (4). Although we hope that vaccines will in the long run end the SARS-CoV-2 pandemic, we will have to continue to live alongside COVID-19 for quite some time. This coexistence also implicates that self-isolation, lockdowns, and other epidemiological measures will have to stay in place or be periodically reintroduced. Although these measures are necessary, the resulting lifestyle changes could in the long run seriously negatively affect public health (5).

This emphasizes the continuing, urgent need for new or repurposed pharmacological treatment options for COVID-19. This treatment should ideally be introduced early in the disease course with an aim to modify its outcome and prevent clinical deterioration. A widespread use of such drugs could reduce hospitalization rates and mortality, allowing a fully outpatient management of COVID-19. Consequently, a reduction in intensive care units occupancy by COVID-19 patients would also reduce the health care costs and allow a better health care resource allocation, which has been an ongoing problem since the start of the pandemic (6). The development of new medicines requires considerable amounts of time and financial resources. It also remains associated with a high risk of failure, as there is no guaranty that the new compound will become an effective, safe, and licensable drug. A more rational approach is drug repurposing, a process that aims to identify new indications for already investigated and approved drugs. This process considerably reduces the time and costs needed for conducting preclinical, phase 1, and phase 2 trials. In the history of medicine, several drugs have been successfully repurposed, with probably the best example being sildenafil. Sildenafil was initially developed by Pfizer as an antihypertensive and later shown to be an effective treatment for erectile dysfunction (7).

Regarding drug repurposing for COVID-19, several drugs have already been investigated. This process, however, is not without its risks, as can be seen from the examples of drugs used early in the pandemic’s course, such as the antimalarial hydroxychloroquine or the antiviral lopinavir, which both have eventually been found to be ineffective (8). Remdesivir, originally developed by Gilead Sciences for use against the Ebola virus, seemed to be a repurposing success story after it received the Food and Drug Administration’s (FDA) approval for use in critically ill COVID-19 patients. Its reported efficacy and justification for use are, however, currently under scrutiny following the release of interim WHO Solidarity trial results (8).

Despite the limited success of drug repurposing attempts for COVID-19 therapy so far, a selective serotonin reuptake inhibitor (SSRI) fluvoxamine has recently demonstrated promising *in vivo* results in preventing clinical deterioration in symptomatic COVID-19 outpatients. This old, widely available, and affordable antidepressant potentially has wide-ranging and important implications for future COVID-19 patient care.

## Emerging evidence for fluvoxamine use in COVID-19 patients

Lenze et al (9) have recently published a double-blind, randomized clinical trial of the effectiveness of fluvoxamine vs placebo in preventing clinical deterioration in symptomatic COVID-19 outpatients in *The Journal of the American Medical Association*. The authors assessed 1337 adults with known or presumed SARS-CoV-2 infection for eligibility, 181 out of whom were included in the age- and sex-stratified randomization, with 152 patient outcomes being included in the final analysis. In the intervention group, 80 patients received 100 mg fluvoxamine three times daily for 15 days following PCR-confirmed SARS-CoV-2 infection, while 72 patients received placebo. In the intervention group, no patient experienced clinical deterioration (defined as onset or hospitalization for dyspnea or pneumonia or a decrease in blood oxygen saturation levels below 92%) compared with 6 patients (8.3%) in the placebo group. This result represented an absolute risk reduction of 8.7% (95% CI, 1.8%-16.4%), which resulted in a significant difference in clinical deterioration between the groups (*P* = 0.009). The intervention group also had a reduction in serious adverse events, with one serious adverse event reported in the fluvoxamine group vs six serious adverse events in the placebo group. Although some may argue that the trial by Lenze et al had inadequate sample size, a strong significant difference observed between the groups and double-blind, randomized design suggest a high level of evidence for off-label fluvoxamine use in clinical practice. According to *clinicaltrials.gov*, the same research group is currently conducting another, significantly larger, randomized, double-blind, multi-center trial aiming to confirm fluvoxamine’s efficacy as an early intervention in COVID-19 patients.

The study by Lenze et al was read by Dr David Seftel, a track physician at the Golden Gates Field Racetrack, where a COVID-19 outbreak occurred a week after the study’s publication. He immediately offered the infected track workers fluvoxamine 50 mg twice daily for 14 days and conducted a prospective cohort study aiming to assess the safety and efficacy of fluvoxamine in newly diagnosed COVID-19 outpatients. His study enrolled 113 infected track workers: 65 in the fluvoxamine group and 48 who refused fluvoxamine treatment. Again, no patients in the fluvoxamine group developed clinical deterioration, while in the no-treatment group 6 (12.5%) patients required hospitalization (10). Until the trial’s results are published, this report may be considered only anecdotal; nevertheless the evidence still strongly supports the use of fluvoxamine in COVID-19 patients.

## Potential mechanism of action of fluvoxamine in COVID-19

**Figure 1:**
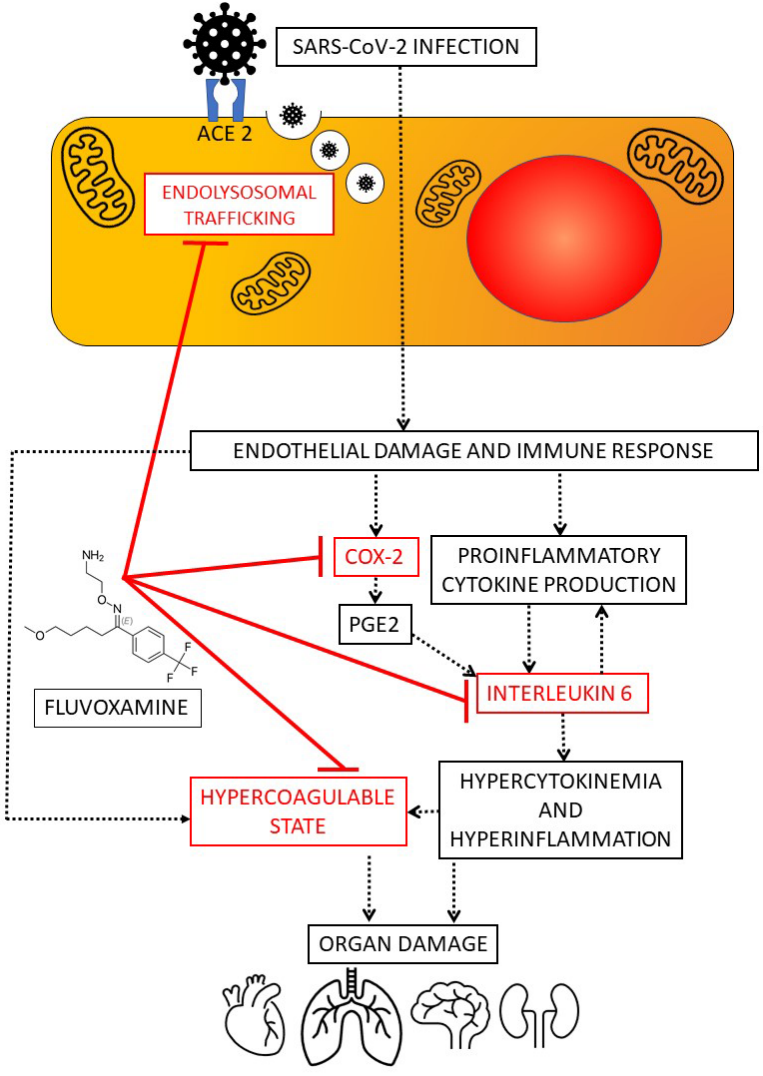
A potential mechanism of action of fluvoxamine in coronavirus disease 2019 pathophysiology. Abbreviations: ACE 2 – angiotensin-converting enzyme 2; COX-2 – cyclooxygenase-2; PGE2 – prostaglandin E2.

The exact mechanism of fluvoxamine’s protective effect in COVID-19 is still partly unknown and is probably multifactorial. The key proposed mechanisms are depicted on Figure 1. Fluvoxamine, next to its antidepressant and anxiolytic effects, seems to possess anti-inflammatory and immunomodulatory properties. As a potential lysosomotropic agent, it could also be capable of influencing endolysosomal trafficking and preventing the hypercoagulative state in COVID-19.

### Anti-inflammatory and immunomodulatory action

A study published in 2019 demonstrated on an animal model of lipopolysaccharide (LPS)-induced sepsis that fluvoxamine had anti-inflammatory properties mediated through its interaction with the sigma-1 receptor (S1R), thus acting protectively against hypercytokinemia (cytokine storm) (11). The same study also found that fluvoxamine significantly reduced the production of interleukin (IL)-6, IL-1 beta, IL-12, and IL-8 induced by LPS in human heparinized peripheral blood.

Other antidepressants also bind to S1R as agonists, and some have shown promise for drug repurposing as anti-SARS-CoV-2 drugs. A preprint of a French multicenter observational retrospective cohort study conducted on 7345 adults hospitalized with COVID-19 reported that SSRI use was associated with a lower risk of intubation or death (12). Among all SSRIs, fluvoxamine possesses the highest affinity toward S1R (13), with positron emission tomography studies demonstrating its high binding capacity at therapeutic doses (14). Fluvoxamine is also known to inhibit cyclooxygenase 2 expression in LPS-stimulated macrophages (15), which by decreasing prostaglandin E2 production can consequently decrease the inflammation-induced IL-6 expression (16). The immunomodulatory effect and IL-6 suppression of fluvoxamine were shown in a clinical study conducted on 30 patients with a mayor depressive disorder. The study demonstrated that at therapeutic doses, fluvoxamine significantly reduced plasma IL-6 levels, which correlated with the drug’s antidepressant effects (17). Recently, it has been revealed that patients who recovered from COVID-19 are at a higher risk of developing psychiatric disorders, especially anxiety and depressive episodes (18). Fluvoxamine could therefore also have a protective effect on mental health in a post-COVID-19 recovery setting. It could exert this effect not only through its anxiolytic and antidepressant action, but also by preventing immune system dysregulation, which could have a role in the development of mental illnesses.

Interleukin 6 is an attractive target with important implications in COVID-19 pathophysiology (19). Namely, the host immune response to SARS-CoV-2 seems to be imbalanced, with low interferon 1 and 3 levels and excessive production of proinflammatory cytokines, especially IL-6 (20), leading to hypercytokinemia, hyperinflammation, and consequently, cytokine storm with acute respiratory distress syndrome (21). The role of IL-6 in COVID-19 is further highlighted by the fact that elevated IL-6 levels in hospitalized COVID-19 patients predict respiratory failure and increased mortality (22,23). Randomized controlled trials conducted with tocilizumab, a humanized monoclonal antibody against the IL-6 receptor, showed inconclusive results in preventing disease progression to intubation or death in hospitalized COVID-19 patients (24,25). It is likely that in any intervention in COVID-19 therapy adequate timing is crucial. A significant advantage of fluvoxamine over tocilizumab as an anti-IL-6 agent might be the possibility of early oral outpatient fluvoxamine treatment, which could prevent the host’s immune imbalance in the later disease course.

A further support for the hypothesis of immune-mediated end-organ damage in severe COVID-19 patients is provided by 30% mortality reduction when a glucocorticoid dexamethasone was used in COVID-19 patients on oxygen therapy or mechanical ventilation (26). If used early in the disease’s course, fluvoxamine could have a synergistic effect with dexamethasone, which could be added later if the patient’s condition deteriorates. This therapeutic combination of early outpatient fluvoxamine and late dexamethasone could additionally decrease patient mortality and should be included in clinical trial protocols.

### Preventing hypercoagulability

Fluvoxamine is an antidepressant of the SSRI class, and its originally off-target peripheral effect on platelet serotonin uptake could potentially also play a role in COVID-19 management. Emerging evidence is pointing to the presence of a hypercoagulable state in COVID-19 and the importance of anticoagulation management (27). Fluvoxamine has a known inhibitory effect on peripheral ^3^H-5HT uptake and has been shown to reduce serotonin concentrations in human platelets and plasma at therapeutic doses. The effect can be observed after just one oral dose of 50 mg, but the reduction was most prominent after six weeks of treatment (-84%) (28). Fluvoxamine could therefore influence the hypercoagulable state in COVID-19 both directly by reducing platelet serotonin levels, but also indirectly through its immunomodulatory effects.

### Potential lysosomotropic agent

Another plausible mechanism of fluvoxamine action in COVID-19 is viral replication inhibition by the disruption of intracellular endolysosomal trafficking. Fluvoxamine has been described as a potential lysosomotropic agent, which means that it can passively diffuse through the endosomal membrane and become protonated and trapped in an acidic pH milieu of the vesicles. This process can change the intravesical pH level, thus potentially interrupting viral fusion and dissemination (29).

## A safe, widely available, and affordable alternative?

Fluvoxamine was first registered in Switzerland in 1983 and is one of the oldest SSRIs still in clinical use. Many post-marketing studies have so far demonstrated its good safety profile. Data from the first 17 years of global post-marketing surveillance, during which 28 million patients were exposed to fluvoxamine, prove that it is a safe and well-tolerated drug in all age groups (30). Even when compared with other SSRIs, fluvoxamine retains a more favorable safety profile as it has a low risk of QT prolongation (31). Unlike other SSRIs, fluvoxamine is also a weak CYP2D6 and a potent CYP1A2 inhibitor (32), but the reported incidence of its drug-associated interactions remains low (33).

Fluvoxamine’s important characteristics considering the SARS-CoV-2 pandemic are its wide availability and affordability. Having at least 15 trade names worldwide, it is highly available (34). The price of one 100 mg oral tablet is US $0.74 (35), which makes the cost of a 15-day treatment as used by Lenze et al only US $33.3. While fluvoxamine is more available and more affordable than other drugs used as standard of care for COVID-19 patients, such as remdesivir or specific anti-spike SARS-CoV-2 monoclonal antibodies, it could lead to a similar or even better treatment outcomes.

For example, remdesivir, which at best shortens the hospitalization time for a median of 5 days (36), and which, according to the WHO Solidarity trial’s interim results, demonstrated no significant impact on survival and duration of hospital stay (8) is priced at US $390 per vial and requires intravenous administration. A five-day treatment with 6 vials of remdesivir therefore costs US $2340 per patient (37), a 70 times higher cost than that of one outpatient fluvoxamine regimen. The high cost of remdesivir is said to be justified by the savings made by an earlier patient discharge, but a widespread use of fluvoxamine early in the disease course could prevent a significant number of hospitalizations, thus not only significantly reducing health care costs but also freeing up health care resources and lowering COVID-19-related mortality.

Moreover, fluvoxamine could also serve as an affordable therapeutic alternative to expensive and poorly available anti-spike monoclonal antibody (mAb) treatments for COVID-19. The FDA has recently approved the emergency use of two monoclonal antibody treatments targeting the SARS-CoV-2 spike protein: bamlanivimab developed by Ely Lilly and the combination of casirivimab and imdevimab developed by Regeneron Pharmaceuticals. The monoclonal antibody treatments have been approved as an early intervention in high-risk patients with mild and moderate COVID-19 and they are believed to speed up recovery time, prevent disease progression, and reduce hospitalization rates (38,39). The US government has already made purchase agreements with both companies – with Ely Lilly for 950 000 vials of bamlanivimab priced at US $1250 per vial, making the purchase worth around US $1.2 billion (40), and with Regeneron for 300 000 doses of casirivimab/imdevimab worth US $450 million (41). In comparison with bamlanivimab and casirivimab/imdevimab, fluvoxamine treatment could have similar or even better results, but it would be respectively around 37 to 45 timesmore affordable per patient treated. Finally, even if new SARS-CoV-2 strains with increased infectivity and mutations affecting the structure of their spike protein become partially or even completely resistant to mAbs therapy, fluvoxamine should remain an effective and safe treatment option.

## Conclusion

We will have to live alongside SARS-CoV-2 for quite some time, and the current therapeutic options unfortunately remain expensive and widely unavailable. Fluvoxamine is among the very few drugs that have demonstrated therapeutic potential and safety profile in a double-blind, randomized clinical trial in humans. In addition, the rationale behind its immunomodulatory and protective effects in COVID-19 is pathophysiologically sound. When comparing the cost of fluvoxamine treatment with the current treatment options, such as remdesivir, bamlanivimab, and/or casirivimab/imdevimab, a full 15-day treatment with fluvoxamine would respectively be 70, 37, or 45 times more affordable per patient. If used early in COVID-19 outpatients, it could prevent many hospitalizations, thus reducing patient mortality, improving allocation of health care resources, and creating significant savings in health care costs. Health professionals and decision makers should become aware of the therapeutic potential of fluvoxamine for COVID-19 patients.
